# Health Literate Schools: Organizational Health Literacy in the School Setting

**DOI:** 10.1093/eurpub/ckac129.249

**Published:** 2022-10-25

**Authors:** S Kirchhoff, O Okan

**Affiliations:** Department of Sport and Health Science, Technical University Munich, Munich, Germany

## Abstract

**Background:**

Fostering health literacy is considered an important objective within school-based strategies of health promotion. To effectively strengthen health literacy in the school setting the approach of organizational health literacy can be transferred to and implemented in schools. This approach combines behaviour-focused measures (providing individual learning opportunities) and environment-orientated measures (changing school's framework conditions). The HeLit-Schools project aims at developing a school-based concept of organizational health literacy and tools to support schools in becoming health-literate schools.

**Methods:**

Existing concepts of organizational health literacy of other setting are analysed and adapted to the school setting. The concept development includes the expertise of actors from the school field, health literacy research and health sector. They contribute by commenting, reviewing and validating the conceptualization at different stages in the development process. A quantitative assessment of schools’ organizational health literacy is planned to further validate the concept. Furthermore, a screening of existing materials and programs for schools to use to foster health literacy is being conducted.

**Results:**

The developed HeLit-Schools concept describes eight standards of a health-literate school. Every standard focuses on a specific area within the school's organization that can be addressed and changed in order to strengthen everyone's health literacy at school more effectively. To additionally support schools in fostering health literacy in school as well as in the classroom, a structured collection of existing (teaching) materials and programs was assembled.

**Conclusions:**

Enhancing schools’ organizational health literacy and thus promoting school development by optimizing schools’ structures, processes and conditions in a health-literate matter can lead to an effective and sustainable strengthening of health literacy of every individual at school.

